# Is 80% satisfaction still the expectation in modern TKA mechanically aligned with robot assist? We think not

**DOI:** 10.1007/s11701-024-01888-9

**Published:** 2024-03-23

**Authors:** Nanchappan Selvanathan, Femi E. Ayeni, Rami Sorial

**Affiliations:** 1 Dept of Orthopaedics, Nepean Hospital, Derby Street, Kingswood, NSW 2747 Australia; 2 Nepean Clinical School, Nepean Institute of Academic Surgery, The University of Sydney, 62 Derby St, Kingswood, NSW 2747 Australia

**Keywords:** Robotic-assisted total knee arthroplasty (TKA) (ROSA), Mechanical alignment, Soft tissue release

## Abstract

Several studies reported that20% of patients were unhappy with the outcome of their total knee arthroplasty (TKA). Having commenced robot assist TKA whilst maintaining the goal of implanting the prosthesis to a neutral mechanical axis, we reviewed our patients to find out if we also have a 20% rate of patients being unhappy with the outcome of their knee replacement surgery. We hypothesized that rate of patient satisfaction would be higher than 90% with robot-surgical assistant (ROSA) technique. The first 175 patients who underwent ROSA TKA were reviewed at a minimum of 1 year postoperatively. All TKAs were performed using ROSA technique with Persona cementless prosthesis aiming to restore neutral mechanical coronal alignment with flexion gap balancing. We investigated whether or not the patients were happy they had their knee replacement surgery and whether they were happy with the outcome. 165 (94%) of 175 patients, were contactable with 1 deceased and 9 uncontactable. From the 165 patients who participated in the study, 95% of patients were happy they had the surgery done and 93% were happy with the outcome of their knee replacement. A sub analysis showed that patients who had simultaneous bilateral TKA were significantly less likely to be happy than staged procedures (*p* < 0.05). Total knee replacement utilising robot technique with modern implants and aiming for mechanical coronal alignment of the implants to restore the mechanical axis with flexion gap balancing may result in > 90% of patients being happy with the outcome of their surgery.

## Introduction

Robotic-assisted TKA was introduced to the Australian market in 2015. Despite this, it remains an emerging technology that some surgeons are using for primary TKA. Knee replacement is a cost-effective and successful treatment for knee arthritis [1] however, 20% of patients are reported to remain unhappy after the procedure [2]. This unhappy 20% is often quoted in many publications and particularly with many scientific presentations and now accepted as a baseline to advise surgeons to use technology and change alignment philosophies to decrease the number of patients who are unhappy with the results of their TKA. The message since the introduction of robot technology is that utilization of robot assist improves the accuracy of intra-operative soft tissue assessment and intra-operative execution of bone cuts and soft tissue balance and lead to better patient satisfaction rates.

The emerging philosophies on alignment are advocating for a change in targets to optimize outcomes and improve kinematics of knee replacements by adopting more kinematic or functional alignment goals. There are many postulations to the causes of an unhappy knee. Aside from the obvious infection, fracture, loosening and gross malalignment, there may be subtle changes in alignment that may be limiting patients’ satisfaction with their new knee and advocates for new alignment targets are predicting significant improvement in the 20% unhappy rate which are usually assessed with a barrage of outcome scores that are employed repeatedly in most clinical practices.

We aimed to establish our own rate of patient satisfaction using the improved accuracy of robot technique but maintaining the same mechanical alignment philosophy before looking at other alignment strategies so that a fair comparison can then be made. However, we have chosen to simplify this right down to a simple question to assess our patient cohort and determine if they are either happy or unhappy with their new knee replacement. Several scoring systems are being used to predict functional outcomes; however, none is considered a gold standard [3]. A good outcome score does not mean patients are truly satisfied and happy with their knees.

Hence, we sought to investigate whether our patients were simply happy with the result of their knee replacements 1 year after undergoing a robot-assisted mechanically aligned TKA by specifically asking them that question. We chose to review the cohort of our first group of patients with robotic technique to identify how they relate to the often-quoted figure of 20% unhappy ratio for knee replacements and expected our rate of happy patients to be higher.

## Methodology

This was a cross sectional study, carried out at two private hospitals where the senior author works. We collected data from all patients who underwent robot-assisted Total Knee Arthroplasty (TKA) between December 2019 (when the first robot was purchased) and August 2021. All patients that were performed at the hospital with the robots had this technique utilised. There were no exclusions. Patients who had a standard conventional knee replacement at a third hospital where there was no robot were not included in this study. All patients included in this study were at least 12 months post surgical procedure. Robot assisted surgery using ROSA (Zimmer Biomet, Warsaw, IN) was used in all surgeries aiming to restore mechanical alignment for a hip-knee-ankle angle (HKA) of 0 with a flexion gap balancing technique using the FuZion (Zimmer Biomet, Warsaw, IN) device. Surgeries were performed by single surgeon using a standard medial parapatellar approach without a tourniquet utilising the cementless Persona (Zimmer Biomet, Warsaw, IN) prosthesis.

The standard surgical exposure was just sufficient to expose the anterior aspect of the tibia to ensure juxtaposition of the tibial cutting block of the robot arm. Any further exposure of the antero medial tibia was subsequently assessed as a medial release. All large and accessible osteophytes were removed. Note, in this group of patients the standard arrays were placed within the standard incision. The precut balancing plan was affected with the robot after soft tissue laxity was incorporated into the balance algorithm, after assessing through the range of motion with varus and valgus loading.

In all cases the femoral and tibial bone cuts were aligned perpendicular to the mechanical axis (0 degrees varus valgus). Balancing equation was aimed to ensure at least 19 mm of joint space in the medial compartment for a valgus deformity or the lateral compartment for a varus deformity. The opposite compartment was accepted at whatever the tighter joint space was observed, as the surgeon would then perform the required soft tissue releases to ensure balance after bone resections and removal of remaining osteophytes. Once the proximal tibial and distal femoral cuts are made and validated the remaining osteophytes were removed and the extension space assessed with the spacer block.

The aim was to ensure sufficient space in one compartment to accommodate the smallest spacer block and ensure extension was between 0 and 10 degrees. If the medial compartment was tight, then a posteromedial release would be completed as needed to affect balance and fully accommodate the spacer. If the lateral compartment was tight, then a fenestrated posterolateral capsular release plus or minus PCL sacrifice completed as needed to affect balance and fully accommodate the spacer. These releases were documented.

The FuZion device would then be used and tensioned in 95 degrees of flexion as required by the ROSA workflow to balance the flexion space by rotating the femoral component position till equal tension and space in both compartments achieved. This rotation would then be incorporated into the flexion balance algorithm. The final flexion balance was confirmed to ensure at least 19 mm medial and lateral joint spaces in flexion before the final femoral bone cuts are completed, dictating the rotation of the femoral component independent of the trans epicondylar or posterior condylar axes.

The trial reduction is the final opportunity to ensure balance is achieved and if further releases are required. This is particularly relevant for sagittal balance and flexion range and if required, the PCL may be fenestrated if any tibial lift off is observed. After implantation of the true prosthesis the final balance through the range is assessed and documented. Patients were mobilised on Day 1 as per standard physiotherapy protocols full weight bearing as tolerated and discharged when independent on crutches. At a minimum of 1 year following the surgery an independent investigator being the current arthroplasty fellow and junior author who was not linked to the surgery or with the patients, called all patients and asked each 2 questions. “Are you happy you had your left/right knee replacement surgery? and “Are you happy with the outcome of your left/right knee replacement?”.

This research has been approved by the authors’ affiliated institutions. All enrolled patients gave their informed consent.

### Statistics

Descriptive statistics was used to summarise outcomes. Fisher Exact test was used where appropriate to test for association between surgical parameters and outcomes. For all analyses, *p* values of less than 0.05 was considered statistically significant. All analyses were performed in statistical program GraphPad Prism Version 9.4.1 (681).

## Results

A total of 175 patients underwent robot-assisted TKA during the study period. One patient had deceased during the study period, while 9 patients were lost to follow-up despite multiple attempts to locate them, resulting in 165 patients (94.3%) being included in this study. From the total group the age range was from 48 to 90 yrs with an average of 71yrs. The most common age group is between 65–74 (44.3%). Most patients were female (74.9%) and 64.9% had an American Society of Anaesthesia (ASA) score of 2. Pre-obese group, BMI from 25–29.99 had the most patients with 31.6% however 58.6% of patients were in the obese group and more than 15% had a BMI > 40 (Table 1).

**Table 1:** Demography of patients included in the study

Age group	< 55	4%
	55–64	24.1%
	65–74	44.3%
	> 75	27.6%
Gender	Male	44 (25.1%)
	Female	131 (74.9%)
ASA score (American Society Anaesthesia physical score)	1	12.6%
	2	64.9%
	3	21.8%
	4	0.6%
BMI (body mass index)	Underweight (< 18.50)	0.6%
	Normal (18.50–24.99)	9.2%
	Pre-obese (25.0–29.99)	31.6%
	Obesity class 1(30.0–34.99)	27%
	Obesity class 2(35.5–39.9)	16.1
	Obesity class 3(> 40)	15.5%

In response to our two questions, 92.7% of the patients reported they were happy with the outcome of their knee replacement and 95.2% reported they were happy they had the surgery (Table 2). A soft tissue release was required for balance in 49 patients (29.7%). Most of the patients had single side knee surgery done, with 8 patients (4.8%) having had simultaneous bilateral knee surgery while12 patients (7.3%) had staged knee surgery within 6 months (Table 2).

**Table 2:** Outcome and Surgical Characteristics among patients (*n* = 165)

		Frequency	Percentage (%)
Happy with the outcome	No	12	7.3
	Yes	153	92.7
Happy had surgery	No	8	4.8
	Yes	157	95.2
Type of surgery	Single	145	87.9
	Simultaneous	8	4.8
	Staged	12	7.3
Soft tissue release	No	116	70.3
	Yes	49	29.7

If a soft tissue release was required for balance of the TKA (49 patients 29.7%), this group of patients at minimum 1 year FU did not demonstrate any significant difference from the group that did not require any soft tissue release (116 patients 70.35) with being happy with the outcome of their knee replacement *p* > 0.05 (Table 3). Patients who underwent single side surgery were significantly associated with a better outcome, being happy with the result of TKA and being happy they had the surgery, compared to patients who underwent simultaneous bilateral TKA, *p* < 0.05. In contrast, there was no significant difference in being happy with the outcome and being happy they had surgery when single side surgery was compared with the bilateral staged TKA (*p* > 0.05).

**Table 3:** Outcome and Surgical Characteristics with soft tissue release among patients (*n* = 165)

		Happy with outcome		*p* value	Happy had surgery		*p* value	
		No	Yes		No	Yes		
Soft tissue release done	No	10	106	> 0.513	7	109	> 0.438	
	Yes	2	47		1	48		
Type of surgery								
Single		6	139	<0.006	4	141	< 0.034	
Simultaneous		4	4		2	6		
Single		6	139	> 0.116	4	141	> 0.068	
Staged		2	10		2	10		

## Discussion

Satisfied patients are happy patients. There are limited studies conducted asking whether patients are happy post-robot-assist Total Knee Arthroplasty (TKA). In this study we asked two objective questions. The first question was whether the patients were happy they had the surgery done? The reason for this question signifies retrospectively whether patients feel it was the right decision they made to have knee replacement surgery considering the level of function they have at 1 year following the procedure. The second question is whether the patients were happy with the outcome of the surgery which would signify if their level of function met their expectations? The two questions are germane and provide objective valuable feedback to the surgeon from the patient’s perspective as to whether the expectation of the surgery has been achieved.

Many studies use the Likert scale satisfaction to measure patient satisfaction post-TKA. Likert scale is a validated measurement that consists of parameters: very dissatisfied, dissatisfied, neutral, satisfied, and very satisfied [4]. Goldsmith et al. [4] reported that 84% of patients were very satisfied and, 8% were neutral and 8% were very dissatisfied [4]. However, the disadvantage of this scale is that there is no clear difference between each parameter [5]. We believe it creates confusion among patients and involves language proficiency. Thus, supporting our methodology of expecting a binary response from our patients, yes or no.

The unhappy knee can be divided into knee-related causes and non-knee-related causes. Diagnosis is critical, especially in knee-related causes before committing to revision surgery. Non-knee related causes are further divided into peripheral causes (referred pain from spine or hip) and psychological causes. It’s been shown that patients with anxiety and depression present with unhappy knees [6, 7]. Patients with a psychological condition would provide poor outcome scores [7]. None of our unhappy patients had any known psychological conditions. The aim of this study was to identify the quantity of happy patients and no attempt was made by the investigator to identify the causes of the unhappy responses as this would be difficult over the phone and would require a consultation and examination. This will form part of a further review. But noting that 4(2%) knees (unilateral) were happy that they had the surgery but not happy with the outcome may reflect that these patients had higher expectations of what TKA can do for them but had no regrets to undergo the surgery.

Furthermore 7% (9 patients) were unhappy following TKA, and of these 3patients (6 knees) received bilateral simultaneous TKA. Patients are routinely followed up at 6 weeks, 6 months, and 5 years. PROMS including the Oxford Knee Score are done pre-op and at 6 months only, but beyond this, it is reported to show no difference and be not cost-inefficient [8]. For this study we have specifically utilized the binary scoring system to counter the often quoted and published 20% “unhappy patient” ratio that opens the discussion to new alignment strategies to improve outcomes. In addition, good PROMS do not necessarily mean patients are satisfied or “happy” with their surgery, although they are a good gauge of patients’ functional improvement pre and post-surgery [9].

We retrospectively checked our robotic data and it showed balanced parameters. These patients have not been booked in for a follow-up and no implant revisions were documented at the surgeon’s portal in the AOANJRR registry. Knee alignment has been extensively researched and is postulated as the cause of 20 percent of unhappy knees [10–12] and thus changing to an alternate alignment philosophy rather than restoring mechanical axis will correct this level of dissatisfaction. In our patients’ cohort, only 7% were unhappy with the outcome of their knee replacement and there is no study we could identify that looked specifically into the rate of unhappy knees among TKA with kinematic alignment philosophies. However, in regard to radiological and functional outcomes, there is no difference between both alignment philosophies [13].

Patient selection is critical in performing simultaneous bilateral TKA. Studies on robot-assisted bilateral simultaneous TKA are limited. Anderson et al. and Song et al. [14, 15] reported no difference in functional outcome scores comparing the conventional instrumented knee and the robotic group for bilateral simultaneous TKA [14, 15]. In our study, for patients who had bilateral simultaneous robot-assisted TKA three patients (6 knees) were unhappy with the outcome of their surgery, and two patients (4 knees) were happy. Compared to six patients (12 knees) for whom we did staged bilateral TKA, all of whom were happy with the outcome of the knee which was significant (*p* < 0.05). we acknowledge these numbers are small but do show a trend that is worth noting and we believe further research is required to compare the two cohorts of staged and simultaneous robot assist TKA with a larger sample group.

In our study, over 70% of patients did not require a soft tissue release as we previously reported for this group [16] and our analysis did not show any significant difference in patients’ happiness whether they required a soft tissue release or not. Abhari et al. [17] reported at 1 year follow up that 93% were satisfied in the kinematic alignment group compared to 83% in the mechanical aligned group using conventional instruments [17]. Similarly, Winnock et al. [18] reported higher outcome scores and satisfaction in the kinematic alignment group compared to mechanical alignment using robot-assisted technique [18]. However, none of these studies were randomized, hence, substantial research is still required in this area.

One of the limitations of this study is the phone conversation method we employed to extract information from the participants as we couldn’t explore further why the patients were unhappy. We also didn’t explore if the participants that were unhappy with the outcome have been unhappy since they had surgery or just of recent onset change. However, the strength of the study is that over 94% of our patients were contactable and agreed to participate in the study.

## Conclusion

Our study showed that 93% of our patients are happy with the outcome of Robot-assisted TKA utilizing mechanical coronal alignment to restore the mechanical axis. We also observed that patients who had bilateral simultaneous TKA were less happy with the outcomes and would suggest in patients presenting with bilateral knee pathology, consideration is given to performing staged procedures where this is feasible.

## Data Availability

The data that support the findings of this study are available from Nepean Blue Mountain Local Health District, but restrictions apply to the availability of these data, which were used under license for the current study, and so are not publicly available. Data are however available from the authors upon reasonable request and with permission of Nepean Blue Mountain Local Health District.

## References

[1] Scott CE, Howie CR, MacDonald D, Biant LC (2010) Predicting dissatisfaction following total knee replacement: a prospective study of 1217 patients. J Bone Joint Surg Br 92(9):1253–1258 20798443 10.1302/0301-620X.92B9.24394

[2] Kahlenberg CA, Nwachukwu BU, McLawhorn AS, Cross MB, Cornell CN, Padgett DE (2018) Patient satisfaction after total knee replacement: a systematic review. HSS J 14(2):192–201 29983663 10.1007/s11420-018-9614-8 PMC6031540

[3] Collins NJ, Misra D, Felson DT, Crossley KM, Roos EM. Measures of knee function: International Knee Documentation Committee (IKDC) Subjective Knee Evaluation Form, Knee Injury and Osteoarthritis Outcome Score (KOOS), Knee Injury and Osteoarthritis Outcome Score Physical Function Short Form (KOOS-PS), Knee Outcome Survey Activities of Daily Living Scale (KOS-ADL), Lysholm Knee Scoring Scale, Oxford Knee Score (OKS), Western Ontario and McMaster Universities Osteoarthritis Index (WOMAC), Activity Rating Scale (ARS), and Tegner Activity Score (TAS). Arthritis Care Res (Hoboken). 2011 Nov;63 Suppl 11(0 11):S208–28. 10.1002/acr.20632 PMC4336550 22588746

[4] Bryan S, Goldsmith LJ, Davis JC, Hejazi S, MacDonald V, McAllister P, Randall E, Suryaprakash N, Wu AD, Sawatzky R (2018) Revisiting patient satisfaction following total knee arthroplasty: a longitudinal observational study. BMC Musculoskelet Disord 19(1):423 30497445 10.1186/s12891-018-2340-z PMC6267049

[5] Klem NR, Kent P, Smith A, Dowsey M, Fary R, Schütze R, O’Sullivan P, Choong P, Bunzli S (2020) Satisfaction after total knee replacement for osteoarthritis is usually high, but what are we measuring? a systematic review. Osteoarthr Cartil Open 2(1):100032 36474554 10.1016/j.ocarto.2020.100032 PMC9718338

[6] Ali A, Lindstrand A, Sundberg M, Flivik G (2017) Preoperative anxiety and depression correlate with dissatisfaction after total knee arthroplasty: a prospective longitudinal cohort study of 186 patients, with 4-year follow-up. J Arthroplasty 32(3):767–770 27692782 10.1016/j.arth.2016.08.033

[7] Hirschmann MT, Testa E, Amsler F, Friederich NF (2013) The unhappy total knee arthroplasty (TKA) patient: higher WOMAC and lower KSS in depressed patients prior and after TKA. Knee Surg Sports Traumatol Arthrosc 21(10):2405–2411 23358576 10.1007/s00167-013-2409-z

[8] Canfield M, Savoy L, Cote MP, Halawi MJ (2019) Patient-reported outcome measures in total joint arthroplasty: defining the optimal collection window. Arthroplast Today 6(1):62–67 32211477 10.1016/j.artd.2019.10.003 PMC7083724

[9] Ackermans L, Hageman MG, Bos AH, Haverkamp D, Scholtes VAB, Poolman RW (2018) Feedback to patients about patient-reported outcomes does not improve empowerment or satisfaction. Clin Orthop Relat Res 476(4):716–722 29406450 10.1007/s11999.0000000000000069 PMC6260089

[10] Rothwell AG, Hooper GJ, Hobbs A, Frampton CM (2010) An analysis of the Oxford hip and knee scores and their relationship to early joint revision in the New Zealand Joint Registry. J Bone Joint Surg Br 92(3):413–418 20190314 10.1302/0301-620X.92B3.22913

[11] Bourne RB, Chesworth BM, Davis AM, Mahomed NN, Charron KD (2010) Patient satisfaction after total knee arthroplasty: who is satisfied and who is not? Clin Orthop Relat Res 468(1):57–63 19844772 10.1007/s11999-009-1119-9 PMC2795819

[12] Baker PN, van der Meulen JH, Lewsey J, Gregg PJ (2007) National Joint Registry for England and Wales the role of pain and function in determining patient satisfaction after total knee replacement data from the National Joint Registry for England and Wales. J Bone Joint Surg Br 89(7):893–900 17673581 10.1302/0301-620X.89B7.19091

[13] Sappey-Marinier E, Pauvert A, Batailler C, Swan J, Cheze L, Servien E, Lustig S (2020) Kinematic versus mechanical alignment for primary total knee arthroplasty with minimum 2 years follow-up: a systematic review. SICOT J 6:18 32553101 10.1051/sicotj/2020014 PMC7301633

[14] Batailler C, Anderson MB, Flecher X, Ollivier M, Parratte S (2023) Is sequential bilateral robotic total knee arthroplasty a safe procedure? a matched comparative pilot study. Arch Orthop Trauma Surg 143(3):1599–1609 35536354 10.1007/s00402-022-04455-9

[15] Song EK, Seon JK, Park SJ, Jung WB, Park HW, Lee GW (2011) Simultaneous bilateral total knee arthroplasty with robotic and conventional techniques: a prospective, randomized study. Knee Surg Sports Traumatol Arthrosc 19(7):1069–1076 21311869 10.1007/s00167-011-1400-9

[16] Selvanathan N, Ayeni FE, Sorial R (2023) Incidence of soft tissue releases in robotic assisted cementless TKA with mechanical alignment and flexion gap balancing. Arthroplasty 5(1):28 37280658 10.1186/s42836-023-00188-1 PMC10245504

[17] Abhari S, Hsing TM, Malkani MM, Smith AF, Smith LS, Mont MA, Malkani AL (2021) Patient satisfaction following total knee arthroplasty using restricted kinematic alignment. Bone Joint J. 103(6):59–66 34053299 10.1302/0301-620X.103B6.BJJ-2020-2357.R1

[18] Winnock de Grave P, Luyckx T, Claeys K, Tampere T, Kellens J, Müller J, Gunst P (2022) Higher satisfaction after total knee arthroplasty using restricted inverse kinematic alignment compared to adjusted mechanical alignment. Knee Surg Sports Traumatol Arthrosc 30(2):488–499 32737528 10.1007/s00167-020-06165-4 PMC8866329

